# Complete response in advanced breast cancer patient treated with a combination of capecitabine, oral vinorelbine and dasatinib

**DOI:** 10.1186/s40164-018-0094-9

**Published:** 2018-01-24

**Authors:** V. Sgroi, M. Bassanelli, M. Roberto, E. Iannicelli, R. Porrini, P. Pellegrini, A. Tafuri, P. Marchetti

**Affiliations:** 1 Department of Molecular and Clinical Medicine, Medical Oncology Unit, “Sapienza” University of Rome, Sant’ Andrea Hospital, Rome, Italy; 2 Department of Radiology, Faculty of Medicine and Psychology, “Sapienza” University of Rome, Sant’ Andrea Hospital, Rome, Italy; 3 Department of Molecular and Clinical Medicine, Hematology,, “Sapienza” University of Rome, Rome, Italy

**Keywords:** Advanced breast cancer, Chronic myeloid leukemia, Dasatinib, Capecitabine, Vinorelbine

## Abstract

**Background:**

Currently, there are no data available on the best choice of treatment in heavily pretreated patients with advanced breast cancer. However, the combination of oral vinorelbine and capecitabine has been demonstrated to be effective and safe in patients with advanced breast cancer pretreated with anthracycline. Furthermore, some studies assessed the activity of dasatinib, an oral tyrosine kinase inhibitor that inhibits five oncogenic tyrosine kinase families, alone or in combination with different chemotherapy in patients affected with advanced breast cancer.

**Case presentation:**

A patient with metastatic breast cancer, hormone receptor positive and human epidermal grow factor receptor 2 negative, pretreated with epirubicine, taxanes and nab-paclitaxel, was submitted to third line chemotherapy with vinorelbine 60 mg/m^2^ on day 1, 8 plus capecitabine 1000 mg/m^2^ twice daily from day 1 to day 14 every 21 days. The patient was taking also dasatinib 100 mg once daily for chronic myeloid leukemia. The treatment was well tolerated and, after 15 months, computed tomography scan showed a complete response of liver metastases and bone stable disease. After another 28 months, a 18-fluorodeoxyglucose positron emission tomography scan showed a metabolic response of bone metastases without other site of disease.

**Conclusions:**

This is the first case in literature about activity of dasatinib in combination with a chemotherapy schedule of oral vinorelbine and capecitabine in advanced breast cancer. This treatment showed both good tolerability and great activity with a long progression free survival of 54 months.

## Background

Breast tumor is the most frequent cancer among women in the 0–49 years age group (41%), meanwhile diagnosis may occurs in 35 and 22% of women in the age groups 50–69 and > 70 years, respectively. Advanced breast cancer (ABC) patients have a 5 years survival rate of about 22% [1]. Currently there are no standard treatment in heavily pretreated ABC and thus, the choice of therapy is mainly driven by patient features, comorbidity, histology and potential toxicities.

We report the case of a patient affected with ABC and chronic myeloid leukemia, who has achieved a complete response during chemotherapy treatment with vinorelbine and capecitabine administered together with dasatinib for the hematologic disease.

She reported a longer progression free survival (PFS) than other patients included in all published clinical study, in which they were treated with dasatinib concomitantly with chemotherapy drugs [10, 16].

## Case presentation

We report the case of a 58-year-old women, actually in post menopausal state, followed in our hospital for ABC, who underwent radical right mastectomy in 1994, when she was 35 year old, for a stage I breast ductal carcinoma, hormone receptor (HR) negative, human epidermal grow factor receptor 2 (HER2) negative. In 1997 a computed tomography (CT) scan showed multiple backbone metastases, confirmed at bone biopsy (breast ductal carcinoma, HR positive, HER2 negative), so she was submitted to a first line chemotherapy with epirubicina 75 mg/m^2^ plus paclitaxel 75 mg/m^2^ 3 weekly for six cycles. The metastases were evident to the CT-scan, so she was not subjected to bone scintigraphy. Thereafter, she underwent a palliative radiotherapy on the 10th dorsal vertebra (30 Gray in ten fractions, 3 Gray for each fraction) and a maintenance anti-hormonal therapy with gonadotropin releasing hormone (LHRH) analogue and letrozole, a non-steroidal aromatase inhibitor (AI), then replaced with exemestane, a steroidal AI for intolerance, until June 2011.

In 2009 a chronic myeloid leukemia (CML) was diagnosed with a osteomedullary biopsy that showed overexpression of 180% of Breakpoint Cluster Region of Abelson gene (BCR-ABL gene). The patient started imatinib, which was suspended in May 2010 for resistance onset. So, she started dasatinib, a potent orally SRC family kinase (SFK) inhibitor, at a dosage of 100 mg daily.

**Fig. 1:**
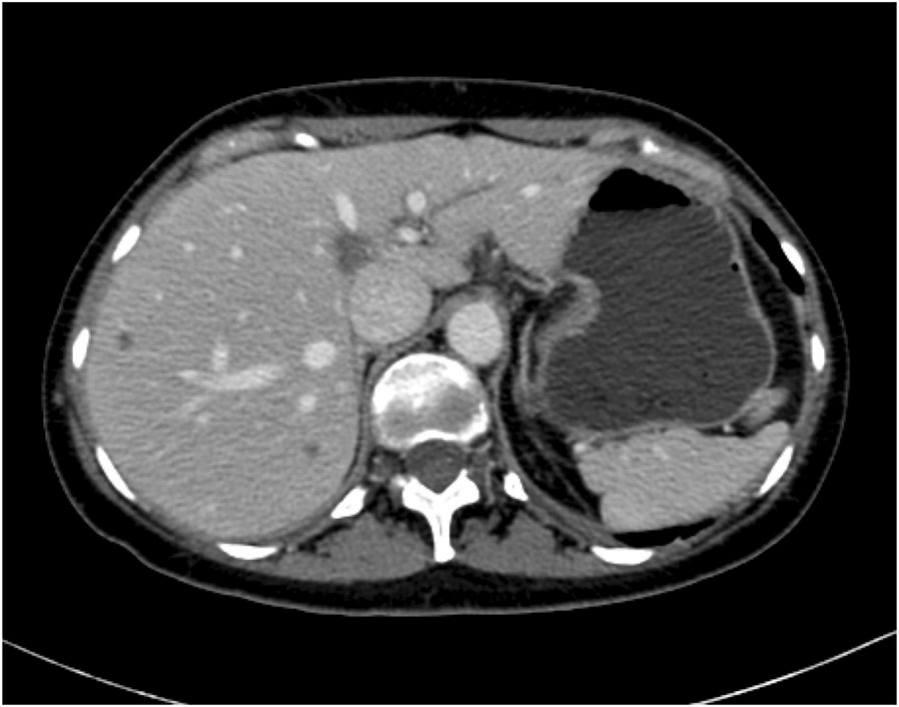
Hepatic lesions. In July 2011, CT scan showed occurrence of hepatic lesions

In July 2011, CT scan showed multiple lesions in different hepatic segments with maximum diameter of 2 cm at CT scan (Fig. 1); so a liver biopsy was performed and the histology confirmed the diagnosis of breast ductal carcinoma, HR positive, HER2 negative.

**Fig. 2:**
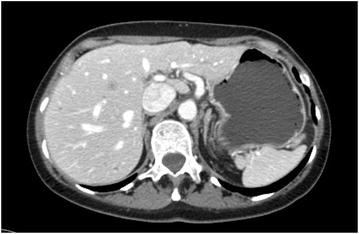
New hepatic lesion. In October 2012, CT scan showed liver progression for the appearance of a new lesion

In August 2011, she started a second line chemotherapy with nab-paclitaxel 260 mg/m^2^ 3 weekly supported with granulocyte colony stimulating factor (GCSF); stopping dasatinib for 6 days during the administration of GCSF, as for hematological counseling. The treatment has been continued for 16 cycles with a stable disease (SD) as a best response. In October 2012, after a total of 14 months of therapy, a CT scan showed liver progression with the appearance of a 11 mm new lesion in IVb segment (Fig. 2), thus she started a third line chemotherapy with vinorelbina 60 mg/m^2^ on day 1, 8 plus capecitabine 1000 mg/m^2^ twice daily from day 1 to day 14 every 21 days.

In March 2013, after eight cycles, the CT scan showed a liver partial response and after another four cycles a further reduction. The major toxicities occurred were grade 2 neutropenia and diarrhea. Hence, capecitabine dosage was reduced by 25%.

**Fig. 3:**
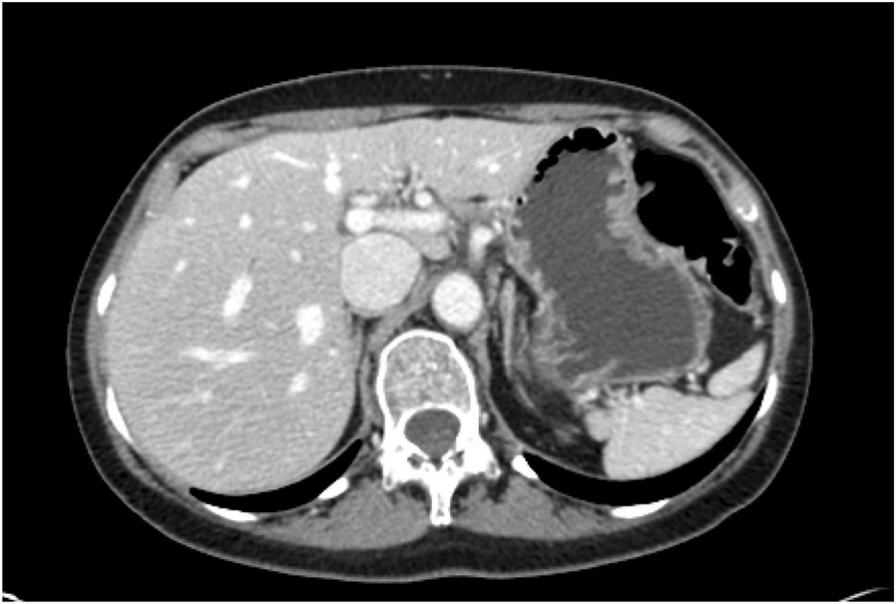
Complete response. In January 2014, CT scan showed a complete response of liver metastases

In January 2014, after 17 cycles, the CT scan showed a complete response of liver metastases (Fig. 3) and bone SD.

In May 2016, after 43 months, a 18-fluorodeoxyglucose positron emission tomography (18-F-FDG PET) scan, performed due to contrast allergy onset, showed a metabolic response of bone metastases without other site of disease.

Currently, these treatments (capecitabine plus vinorelbine and dasatinib) are still ongoing with a PFS of 54 months and good tolerance.

The CML is in complete molecular response and the molecular examination of peripheral blood showed no expression of BCR-ABL gene.

## Discussion

We discuss the case of a patient affected by a HR positive ABC and CML, who achieved complete tumor response and leukemia remission with the combination of vinorelbine plus capecitabine third line chemotherapy and dasatinib.

Following the appearance of liver metastases, our patient, already being treated with dasatinib for CML, has been subjected to second line chemotherapy with nab-paclitaxel 260 mg/m^2^ every 3 weeks, continuing dasatinib. In the registrative trial, time to progression (TTP) for patient who received in first line nab-paclitaxel compared with paclitaxel was 23 weeks, in second or greater line was 20.9 weeks, but for our patient was of 51 weeks [2].

Currently there is not a standard of care in third line ABC treatment, though eribulin could represent a valid therapeutic option. In the EMBRACE trial the overall survival (OS) was significantly improved in eribulin arm (median 13.1 months, 95% CI 11.8–14.3) when compared with treatment of physician’s choice (10.6 months, 9.3–12.5; HR 0.81, 95% CI 0.66–0.99; p = 0.041) but neutropenia occurred in 52% of subjects in eribulin group [3]. Thus, considering the risk of haematologic toxicity and the history of CML, we excluded this therapy.

Many studies showed the activity of oral vinorelbine and capecitabine in ABC setting [4–6].

Finek et al. described the efficacy and the safety of vinorelbine, 60 mg/m^2^ day 1, 8 every 3 weeks and capecitabine, administrated at dose 1000 mg/m^2^ twice daily from day 1 to 14 every 3 weeks, in anthracycline pretreated patients with ABC. Neutropenia grade 3–4 occurred in only 5.2% of patient. The median PFS and OS was 10.5 and 17.5 months, respectively. The combination appeared particularly effective against life-threatening metastases, with a response rate of 56.5% on liver and lung metastases [7].

Furthermore, Jones et al. showed the clinical activity of oral vinorelbine and capecitabine in 40 patients with ABC: median PFS and OS were 3.4 and 11.3 months, respectively [8].

Therefore, we chose vinorelbine plus capecitabine as third line chemotherapy, carrying on dasatinib administration for CML.

Dasatinib is an oral tyrosine kinase inhibitor that inhibits five oncogenic tyrosine kinase families: BCR-ABL, SRC, c-KIT, EPHRIN receptor A2 (EPHA2), and platelet derived growth factor receptors beta (PDGFR-beta). Of these, particularly SRC are involved in the regulation of cellular proliferation, survival and metastatic ability of cancer [9].

SRC is involved in metastatization process and is frequently overexpressed in human breast cancer [10]. Moreover SRC is important for activation of osteoclasts and late-onset bone metastases, for normal cell growth regulation, angiogenesis, steroid receptor activation, and cell survival.

Consequently, the proliferation of bone metastases from breast cancer should be prevented by inhibiting the SRC activity [11].

Several studies showed an action of dasatinib in tumor control and palliation of bone metastases from ABC. Data about its activity in combination with chemotherapy were extrapolated by previous study in which dasatinib was administrated alone or in combination with capecitabine or paclitaxel [12–14].

In phase II trial of Mayer et al. dasatinib, administered at dose of 100 mg daily, showed a limited activity in patients with ABC both HER2 and/or HR positive subtypes [15]. The most common adverse events (AEs) of any grade were fatigue, gastrointestinal symptoms, headache, pleural effusion, and rash, meanwhile grade 3–4 AEs where diarrhea (9%) and pleural effusion (9%). The objective response rate was 4.3%. Three patients had a partial response lasting 18, 23 and 31 weeks.

The safety of dasatinib in combination with capecitabine for ABC was evaluated in CA180004 trial, in which dasatinib 100 mg once daily plus capecitabine 1000 mg/m^2^ twice daily from day 1 to day 14 every 21 days were tolerable and safe [16]. The most frequent AEs were any grade nausea (58%), hand-foot syndrome (44%) and diarrhea (33% all grade, 8% grade 3–4). However, 43.3% of patients reduced the dose of capecitabine for toxicity and the median PFS was 14.4 weeks. Median PFS in all patients treated was 13.3 weeks, and there are not data on ORR or OS.

Our patient has well tolerated the treatment with dasatinib 100 mg daily and vinorelbine 60 mg/m^2^ day 1, 8 plus capecitabine 1000 mg/m^2^ twice daily from day 1 to day 14 every 21 days for 10 cycles. Within the 10–11th cycle the emergence of both grade 2 neutropenia and diarrhea has led to a 25% reduction in capecitabine dosing. Nevertheless, the patient achieved complete tumor response on CT imaging and a complete metabolic response on 18-FDG PET. To date, the treatment is still ongoing with a patient reported PFS of 54 months.

## Conclusions

To our knowledge, this is the only piece in literature studying the treatment with dasatinib together vinorelbine plus capecitabine. Such combination has proven to act safely and effectively, with an OS of about 5 years. Therefore, more data are waited to confirm the activity of dasatinib in combination with vinorelbine and capecitabine in ABC.

## Abbreviations

HR: hormone receptor
HER2: human epidermal grow factor receptor 2
CT: computed tomography
LHRH: gonadotropin releasing hormone
AI: aromatase inhibitor
CML: chronic myeloid leukemia
BCR-ABL gene: Breakpoint Cluster Region of Abelson gene
SFK: SRC family kinase
EPHA2: EPHRIN receptor A2
PDGFR-beta: platelet derived growth factor receptors beta
GCSF: granulocyte colony stimulating factor
SD: stable disease
18-F-FDG PET: 18 fluorodeoxyglucose positron emission tomography
OS: overall survival
ABC: advanced breast cancer
PFS: progression free survival
TTP: time to progression
AE: adverse event
